# The experiences of family caregivers in response to a dementia diagnosis disclosure

**DOI:** 10.1186/s12888-022-04126-4

**Published:** 2022-07-15

**Authors:** Miao-Chuan Chen, Hung-Ru Lin

**Affiliations:** 1 Department of Nursing, Chang Gung University of Science and Technology, Taoyuan, Taiwan; 2 School of Nursing, National Taipei University of Nursing and Health Sciences, No.365, Ming Te Rd, Pei Tou, District, Taipei, 11219 Taiwan

**Keywords:** Diagnostic disclosure, Dementia, Family caregiver

## Abstract

**Background:**

Dementia is a serious disease that can lead to disability because it impacts the individual’s memory, cognition, behavior, and capacity to perform activities of daily living. While most people prefer to receive a full diagnostic disclosure, the actual care requirements of family caregivers of persons with dementia are often unknown after a dementia diagnosis is disclosed. The primary aim of this study was to explore the experiences of family caregivers in response to a dementia diagnosis disclosure and analyze the care needs of caregivers.

**Methods:**

A qualitative study conducted in accordance with COREQ guidelines. The grounded theory approach was undertaken in 20 family caregivers of persons with dementia, who were selected using purposive sampling. Data were analysed using the constant comparative method.

**Results:**

The core category of this study was "diagnostic disclosure: Start the long road of care challenges", which was defined as describing the experiences of family caregivers of persons with dementia after first being informed of diagnosis. Five major categories describing the experiences of family caregivers following a dementia diagnosis was developed: ‘deciding to seek medical attention,’ ‘the moment of disclosure,’ ‘conveying information,’ ‘maintaining the persons’ functioning,’ and ‘receiving support and living well with dementia.’ Subcategories within each major category also emerged.

**Conclusions:**

Clear diagnostic disclosure is important for ensuring that positive developments can occur in response to disclosure. Healthcare professionals must develop strategies to prevent disclosure from triggering overreactive emotions from persons with cognitive impairments, assist them in understanding their illness in a tactful manner, and ensure that they understand how to cooperate in any subsequent care plans.

## Background

Dementia is a serious disease that can lead to disability because it impacts the individual’s memory, cognition, behavior, and capacity to perform activities of daily living [1]. A World Health Organization (WHO, 2018) survey reported that approximately 50 million people live with dementia worldwide, which is projected to double over the next 20 years and triple by 2050, at which time an estimated 152 million people will be living with dementia. The global population of people living with dementia is growing at a rate of 9.9 million individuals each year, equivalent to a new case being diagnosed every three seconds [2]. As of 2020, 1 in every 77 people in Taiwan is estimated to be living with dementia [3]. Dementia has significant economic implications and is associated with a substantial care burden for the individual, their family, and the community. The increasing rate of dementia diagnoses is likely to be associated with a high societal cost, and the accelerated growth of this population represents a threat to global societies and healthcare systems [1].

In an Australian study, Mastwyk et al. found that 92% of individuals with memory impairment pre-diagnosis wanted to be informed of their diagnosis, with 88% of family members supported this viewpoint [4]. A study conducted in North Wales showed that 94.8% of study participants would prefer to be informed about their diagnosis and a majority of them (84.1%) wanted to be informed about it jointly with their family members [5]. Zou et al. found that 95.7% of caregivers indicated a desire to know about their own diagnosis if they developed dementia, and 82.9% of caregivers would prefer it if the family member who has dementia knows that they have dementia [6]. Those caregivers who argued against diagnostic disclosure believed that knowing about dementia would only make the patient feel uneasy, with no beneficial effects [7]. Other reasons that family caregivers provided for choosing not to disclose a dementia diagnosis included preventing the patient from becoming upset or experiencing difficulty understanding the diagnosis, perceiving the diagnostic disclosure as unnecessary, or following the recommendation of a doctor [8].

Dementia affects both the patient and their family members because most patients are cared for by family members and depend on their assistance and support for individual care and family activities [9]. Most caregivers experience stress, loneliness, and social rejection during the caregiving process. Compared with family caregivers caring for patients without dementia, those who care for patients with dementia often report more negative impacts [10].

What are the experience of family members of persons with dementia in terms of diagnostic disclosure? Few studies to date have explored the intrinsic experiences and needs of family members of persons with dementia following disclosure of dementia diagnosis. The primary aim of this study was to explore the experiences of family caregivers of persons with dementia in response to a dementia diagnosis disclosure, and the care needs of family caregivers at the point of dementia diagnosis is considered.

## Methods

### Study design

This study employed grounded theory method and was undertaken according to the consolidated criteria for reporting qualitative research (COREQ), to explore the feelings and thoughts experienced by family members of persons with dementia following disclosure of the personal dementia diagnosis. The frist author (M-C) consolidated information collected through a review of literature, clinical experiences, and discussions with experts to develop a set of semi-structured interview guidelines (see Table 1). A preliminary study was conducted to verify the appropriateness of the interview guidelines before recruiting participants.

**Table 1:** Interview guidelines for family caregivers

1. Under what circumstances did you learn that your family member was diagnosed with dementia?
2. Do you wish that the doctor would fully disclose your family member’s diagnosis and condition to them? What are the reasons for your answer?
3. How did you feel when the doctor informed your family member about their condition?
4. How do you feel about your family member’s condition?
5. How did your life change after your family member became ill? How did you view or handle these impacts?
6. How did the interactions between you and your family members change when they became ill?

### Setting and participants

A theoretical sampling method was employed in this study, that involves selecting participants based on specific characteristics. Theoretical sampling is a process of data collection for generating theory whereby the analyst jointly collects codes and analyses data and decides what data to collect next and where to find them, in order to develop a theory as it emerges [11]. Therefore, family caregivers of persons with dementia were recruited from the outpatient department of a hospital in northern Taiwan. The inclusion criteria for family caregivers were as follows: (1) currently caring for a family member with dementia; (2) able clearly to communicate in Mandarin or Taiwanese; (3) gave their consent to willingly participate in the study; and (4) not a hired caregiver.

A total of 20 family caregivers of persons with dementia showing willingness to participate in the study, aged 41–83 years (17 females and 3 males, mean age of 61.6 years), completed the interviewes (see Table 2).

**Table 2:** Participants’ basic information

Participants’ code	Gender	Age	Relation	Marital status	Occupational status
C1	Female	69	Wife	Married	Unemployed
C2	Male	62	Son	Married	Full time
C3	Male	68	Son	Married	Full time
C4	Female	70	Daughter	Widowed	Unemployed
C5	Male	68	Son	Married	Unemployed
C6	Female	53	Daughter	Unmarried	Full time
C7	Female	54	Daughter-in-law	Married	Full time
C8	Female	44	Daughter	Unmarried	Full time
C9	Female	57	Daughter-in-law	Married	Full time
C10	Female	56	Daughter	Married	Unemployed
C11	Female	71	Wife	Married	Unemployed
C12	Female	63	Daughter	Married	Unemployed
C13	Female	69	Wife	Married	Unemployed
C14	Female	54	Daughter	Unmarried	Unemployed
C15	Female	41	Grand-daughter	Divorce	Unemployed
C16	Female	65	Daughter	Married	Unemployed
C17	Female	83	Wife	Married	Unemployed
C18	Female	55	Daughter	Unmarried	Unemployed
C19	Female	65	Wife	Married	Unemployed
C20	Female	65	Daughter	Unmarried	Unemployed

### Ethical considerations

This study was approved by the institutional review board of a regional hospital in northern Taiwan (No. 201701078RINC). Prior to conducting the interviews, the researcher clearly explained the participants or their rights, including that they could choose to withdraw from the study at any time without affecting their care or other reprisals, and obtained their written consent.

### Data collection and analysis

Data were collected through individual in-depth, semi-structured interviews. The in-depth interviews are conducted in participant’s native language, either in Mandarin or Taiwanese, without any translation during the process. Potential participants who were referred by attending physicians, and were contacted by the researcher to discuss the details of the study. The participants’ comfort and privacy were considered throughout this study. Data were collected by conducting one-on-one, face-to-face, in-depth interviews in the hospital’s consulting rooms or at participants’ homes. All interviews were held by the frist author (female Registered Nurse, PhD), and who explained the purpose of the study and the estimated length and methods for the interview, assured the participants of their confidentiality throughout the study, and obtained the participants’ consent before conducting the interviews. Each interview was recorded by a digital voice recorder and lasted for 40 to 50 min. Each participant was interviewed just once. Data collection was continued until data saturation was reached. The interviews were immediately transcribed to ensure the completeness of the data.

The authors, who were well trained in qualitative study, conducted data analysis of the verbatim text independently and then cross-examined the analyses together. The constant comparison method was used to analyze the transcripts [12], and open coding began with the first interview. The data were examined line by line to construct the substantive codes. As the data accumulated, early codes were modified and more codes were added. Then, categories were established by grouping similar codes. Through the process of comparison, the categories were reduced and a core category emerged. Finally, a framework was constructed through a continuous modification and integration. New interview data were continuously and repeatedly fit into the categories, and no more new information was added after the participants’ interviews. During the data analysis, the literature on family caregivers in response to a dementia diagnosis disclosure was reviewed to ensure the completeness of the description.

### Trustworthiness

This study adopted the four criteria of credibility, dependability, confirmability, and transferability that were proposed by Lincoln and Guba to evaluate the collected data and ensure the rigor of the analysis [13].

#### Credibility

 There was no relationship between the participant and the researcher prior to the study. Under the guidance of an attending physician, the researcher interacted with the participants during their outpatient treatment to prolong engagement and build trust with the participants. The in-depth interviews began with open-ended questions. The setting for interview were considered to ensure that the participants felt free to express themselves and confirm their intended meanings.

#### Dependability

The data collection and data management was performed by the researcher alone. The data analysis process involved the continuous, repeated, and detailed reading of the transcribed interview contents. The opinions, settings, and overviews of the interactions that occurred during the interviews were recorded to allow others to validate and confirm the consistency of the data. The researcher continuously and repeatedly performed data analysis, induction, and verification to ensure reliability.

#### Confirmability

 The researcher used a field note to engage in constant self-reflection and adjusted, and maintained an objective and neutral stance throughout the study through feedback from professional experts and peers.

#### Transferability

The participants in this study were family caregivers of persons with dementia who were willing to share their true experiences regarding the effects of the disclosure of a dementia diagnosis on their lives. The member checks strategy was conducted that the researchers returned the fundamental structure statement to the participants to ask whether it captured their experiences.

## Results

**Fig. 1:**
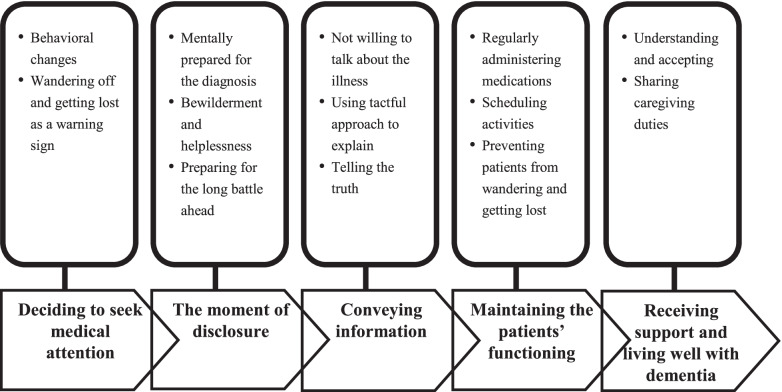
Diagnostic disclosure –Start the long road of care challenges

Based on the data, a framework of the experiences of family caregivers following a dementia diagnosis was developed (Fig. 1). The core category of this study was "diagnostic disclosure: Start the long road of care challenges", which was defined as describing the experiences of family caregivers of persons with dementia after first being informed of diagnosis. This framework comprises the processes through which caregivers attempted to identify strategies for delaying disease-related deterioration, adjusted the interactions with persons with dementia, and shared the experience of accepting and living well with the disease.

Under the framework, five major categories describing the experiences of family caregivers following a dementia diagnosis was developed: ‘deciding to seek medical attention,’ ‘the moment of disclosure,’ ‘conveying information,’ ‘maintaining the personal functioning,’ and ‘receiving support and living well with dementia.’ Subcategories within each major category also emerged.

### Deciding to seek medical attention

The caregivers described deciding to seek medical attention for the persons with dementia after becoming aware of the persons’ abnormal behaviors.

#### Behavioral changes

The caregivers became aware that the persons with dementia were exhibiting unusual behaviors, such as forgetting where they had placed an item they were looking for, asking when mealtime would be after eating, or losing money and suspecting family members of theft. Such as C1 described, “[the person with dementia] kept asking me whether he had eaten, so I felt something amiss about him”.

*During the conversation, he began to become unreasonable, and things would disappear. He is looking for things every day.…….He used to carry a lot of money, necklaces and other things, but he often lost them and couldn’t find them.* (C3)

#### Wandering off and getting lost as a warning sign

Many caregivers described suspecting that something was amiss when their family member lost their way home. Such as C5 said, “At first he lost his way and denied it. He had already lost his way three times before the diagnosis”. And C17 said, “[the persons with dementia] went missing after I went to fetch the medications”.

#### Seeking information

The caregivers described turning to the Internet to seem information regarding their suspicions and hoping to find answers from healthcare professionals. For example:

*I think she should not only be degenerate, because we have also had the opportunity to talk to other older people, but it’s a bit different.…….I looked up information online by myself and found that there are two major types of dementia—Alzheimer’s disease and vascular dementia. But I had to ask the doctor which of the two my family member was diagnosed with.* (C10)

### The moment of disclosure

At the time of the diagnostic disclosure, caregivers had some thoughts and emotional responses, eg: anxious, doubtful, uncertain.

#### Mentally prepared for the diagnosis

Prior to seeking medical attention, some caregivers already suspected that their family members had dementia and were, therefore, unsurprised by the diagnosis. Such as C2 described, “Older people have declining cognitive functions, which differs for everyone. To me, this is normal, so I feel indifferent about it. Everything declines as you age”. And C5 said, “I was mentally prepared. I knew that there was a high chance that the person with dementia would have dementia at that age”.

#### Bewilderment and helplessness

Some caregivers became anxious about the expected progression of dementia and the confusing behaviors of their family members. They described feeling helpless when they think about the long caregiving journey ahead. C11 stated, “At this point, I don’t know what I should do next. At the beginning, it was total confusion, like a drowning person who is unable to reach out to their rescuer”.

#### Preparing for the long battle ahead

After the caregivers came to terms with the lengthy and irreversible nature of dementia, they prepared themselves for the long battle ahead. For example:

*As a family member, when I was informed of the diagnosis, I knew I had to prepare myself for the long, challenge-riddled journey ahead. The attending doctor also told us that the progression of his disease was slow, but it was irreversible!……Compared with other diseases, the course of dementia is very long. For example, my mother had cancer before and passed away in half a year. In fact, the hard work is very short-lived, but family members with dementia have to take care of the person with dementia for a long time. , This is a great responsibility..* (C6)

### Conveying information

After being informed of the diagnosis, family caregivers wondered how to convey the dementia diagnoses to the person with dementia.

#### Not willing to talk about the illness

Some of the caregivers were worried that the persons with dementia would not be receptive to the diagnosis and would feel distressed upon learning of the diagnosis. To them, ignorance is bliss. C2 said, “She [the person with dementia] feels that she’s very strong, so it’s pointless to tell her. She won’t accept the truth because she feels good about herself!”. And C18 stated, “I’m not sure how she really feels. But to me, I think it’s best to not increase her psychological stress”.

#### Using tactful approach to explain

When responding to inquiries from the persons with dementia about their illness, some caregivers took a tactful approach by not fully informing the persons about their condition. C9 said, “She’s always curious about her condition, but I don’t think she is able to accept the truth. I decided to give her a chance by giving hints along the way”.

#### Telling the truth

For some caregivers, avoiding any delay in the opportunity to seek care or establishing care routines was critical. They expected the person with dementia to cooperate after being informed of their diagnosis. For example:

*I really want to tell him about the diagnosis so that he can cooperate with me after understanding his condition. When I tell him to take his medication, he will ask why he needs to take so much of it and wants to withdraw. Therefore, I want the doctor to tell him clearly about his illness and have him accept the truth. Otherwise, he keeps attempting to withdraw from taking his medication.* (C1)

### Maintaining the personal functioning

Although the caregivers were generally aware that no cure exists for dementia, they expressed a desire to delay progression and maintain normal functioning in persons with dementia.

#### Regularly administering medications

The caregivers expected the persons with dementia to be able to adhere to their medication regimens to delay the deterioration of their condition. Such as C11 described, “I want to give my family any medication that could delay their deterioration”. C20 said, “She should take any medication that could alleviate her changes”.

#### Scheduling activities

Some caregivers hoped that they could help the persons with dementia delay the onset of social and functional decline by encouraging participation in social activities. For example:

*I have to find ways to stimulate him. Sometimes it’s a gathering with friends. I learned to play card games myself, and I accompany him to card games, teach him how to play, and ask some friends over to play.* (C8)

#### Preventing persons with dementia from wandering and getting lost

The caregivers used tools, such as identity bracelets or keeping records of the patients’ fingerprints to facilitate the rapid identification of persons with dementia in case they wandered and became lost. C14 said, “We kept thinking about how we can prevent her from getting lost, and we decided to give her a GPS tracker”. C15 also said, “Of course I’m worried about him getting lost. I gave him a bracelet so he can easily be tracked down”.

### Receiving support and living well with dementia

The caregivers were better able to identify with resignation and struggles of persons with dementia once they understood the disease, which helped them change their mindset and accept their family members’ fate.

#### Understanding and accepting

Once the caregivers understood the changes that can occur as dementia progresses, they gradually began to empathize with their abnormal behaviors, which they cannot control. The caregivers began to change their mind and learned to accept reality. C16 said, “I don’t think he wants it to be like this. He doesn’t know exactly why he ended up like this”. C11 said, “Sometimes he throws a tantrum, so now I try to appease him”. Another for example:

*I think I can accept her for what she has slowly become. One reason is that she didn’t do this on purpose, and another is that this disease is irreversible, so I really have to coexist with her current state anyhow. (C12)*

#### Sharing caregiving duties

The caregivers reached a consensus with their family members in terms of caregiving duties. C10 said, “It’s important to share caregiving duties because everyone needs to get the necessary support when in need”. And C5 stated, “I laid out a lot of plans and asked my siblings to cooperate with me”.

## Discussion

The core category of this study was "diagnostic disclosure: Start the long road of care challenges", which comprises the processes through caregivers attempted to identify strategies for delaying disease-related deterioration, adjusted their interactions with one another, and shared the experience of accepting and living well with the disease. This finding was similar to Pesonen et al., their core category of “shared processes in the family” [14]. The diagnosis of dementia was shown to be a mutual turning point for the family, which was experienced and responded to in shared processes within the family.

The caregivers were generally aware of the changes in their family members’ behaviors, which prompted them to seek treatment for the affected family member and attempt to identify the causes of the disease. The affected family member "getting lost" was one event that prompted caregivers to choose to seek professional assistance. This finding was similar to the findings of Bunn et al., as it often requires a triggering event or tipping point for deciding to seek professional help [15]. Therefore, identifying symptoms and seeking professional assistance are regarded as shared processes among the family [14]. A majority of the caregivers seem to have predicted the diagnostic disclosure, as Mastwyk et al. stated, they expressed a desire to seek confirmation [4]. This finding was similar to that reported by Derksen et al., who stated that caregivers were still gathering information to assist with adjustment, as most were concerned that the diagnosis would be dementia [16]. Once the caregivers understood the long-term disease course and irreversible nature of dementia, they would prepare themselves to cope with the disease as long as possible. This is related to the fact that most persons with dementia are cared for by their family members and depend upon them to assist and support their activities of daily living [17]. The disease itself is regarded as both a personal experience for the person with dementia and an issue that the whole family must cope with because a family’s equilibrium is disrupted when a family member is diagnosed with dementia [14].

The caregivers often do not wish for their affected family members to be informed of their diagnosis. This finding is in accordance with those reported by van den Dungen et al. and Mitchell et al., who found that caregivers sometimes chose not to disclose their family members’ diagnosis to them to prevent unease or anxiety and because they regarded the disclosure as being pointless [18, 19]. In the present study, some caregivers attempted a tactful approach to convey information regarding dementia to the persons with dementia without persuading them to understand the disease completely. As noted in the study by Zou et al., "dementia", traditionally used in Chinese, is considered insulting, and caregivers who chose this approach appeared to be describing the situation as a “cognitive impairment” to avoid impulsive reactions from their family members [6].

The family caregivers attempted to help the persons with dementia comprehend their current condition and prognosis in the hope that understanding would enable to cooperate in their follow-up care plans. This finding supports the findings reported by Mastwyk et al., in which caregivers expressed the desire that their family members understood their condition so that treatment plans could be devised and offered to them, and the patients could learn coping strategies [4]. The strategies adopted by the caregivers to delay deterioration in their family members’ conditions include routine medication administration, scheduling activities, and preventing persons with dementia from wandering around and getting lost. These strategies support the findings of van der Roest et al., who reported that caregivers took their own initiative to find information about the disease and sought solutions [20]. Coping strategies included managing their everyday activities; keeping a list of important items; using cues and clues; writing reminders in a diary, notebook, or calendar; promoting their health; engaging in memory training sessions; and maintaining their activity levels [21–24].

The caregivers were better able to identify with the feelings of resignation and struggles with the disease in persons with dementia when they had a better understanding of the disease. Caregivers often chose to accept the patients’ fate and attempted to change their own mindsets to help their family members live well with dementia. This result is consistent with research finding by Derksen et al., who argued that throughout the process of adapting to their roles, some caregivers have been able to better understand the remaining capacities of person of dementia [16]. This approach reflects the values and beliefs of Asian people. The participants in this study are all Taiwanese, who believe that everything that happens to their corporeal form is a matter of fate and view acceptance as the best way to live with things that cannot be changed. Therefore, the family members were compelled to accept the dementia diagnosis and take on the caregiver role, as shown by Mok et al. [25]. Tao et al. also stated that caring for the sick is considered a moral obligation of family members [26]. The caregivers changed how they interacted with the persons with dementia, complied with their needs, and strengthened their relationships during the process. A diagnostic disclosure not only prompts caregivers and persons with dementia to rethink and adjust their interactions to identify effective coping measures but also allows them to recognize the value of their relationship [14]. However, caregivers also felt the burden of their responsibilities, which they wished they could share with others. During this process, they accepted both that their family member was diagnosed with dementia and that their family member’s illness was part of their lives.

This study employed a retrospective interview technique, which can be limited by the participants’ inability to completely recall past experiences. Therefore, the authenticity of the retrospective data remains incomplete. To overcome this problem, a longitudinal study can be conducted in the future, in which data is collected at multiple time points after diagnosis. Such an approach would reveal additional details regarding the participants’ actual life experiences and reveal the changes that occur in response to the use of various coping strategies. In addition, the primary diagnoses of persons with dementia cared for by the participants in this study were Alzheimer's disease and vascular dementia, and they were typically older individuals (mean age: 85.6 years, standard deviation; 5.40 years). Therefore, these results may not be generalizable to persons with other forms of dementia. Subsequent studies can target younger populations or those with early-onset dementia and compare differences in their experiences with dementia diagnosis disclosure. This study did not include some basic information, such as work status, the time since diagnosis, etc., so that we cannot deeply understand their impact on the lives of caregivers.

## Conclusion

This study describes the experiences of family caregivers of persons with dementia in response to being informed of the dementia diagnosis. The core category of this study was "diagnostic disclosure: Start the long road of care challenges". The caregivers accompanied the persons with dementia to seek medical attention and addressed the impacts of the disease from the moment of disclosure. Following the diagnostic disclosure, the caregivers had a clearer direction of how they should adjust themselves to live well with dementia. Changes began once the persons with dementia and caregivers were informed of the diagnosis and accepted it. Despite feeling overwhelmed, the caregivers accepted the reality that their family members were diagnosed with dementia and accepted the role of long-term caregiving. Therefore, clear diagnostic disclosure is important for ensuring that positive developments can occur in response to disclosure. Healthcare professionals must develop strategies to prevent disclosure from triggering overreactive emotions in persons with cognitive impairments, assist them understand their illness in a tactful manner, and ensure they understand how to cooperate in any subsequent care plans.

## Data Availability

The dataset analysed in the current study are available from the corresponding author on reasonable request. The data are not publicly available due to the information could compromise research participant privacy.
